# Preclinical dental students self-assessment of an improved operative dentistry virtual reality simulator with haptic feedback

**DOI:** 10.1038/s41598-023-29537-5

**Published:** 2023-02-17

**Authors:** Pedro Rodrigues, Francisco Nicolau, Martim Norte, Ezequiel Zorzal, João Botelho, Vanessa Machado, Luís Proença, Ricardo Alves, Carlos Zagalo, Daniel Simões Lopes, José João Mendes

**Affiliations:** 1 Clinical Research Unit (CRU), Egas Moniz Center for Interdisciplinary Research, Egas Moniz School of Health and Science, 2829-511 Almada, Portugal; 2 Egas Moniz Center for Interdisciplinary Research, Egas Moniz School of Health and Science, 2829-511 Almada, Portugal; 3 Instituto Superior Técnico, Universidade de Lisboa, Lisbon, Portugal; 4 INESC ID, Lisbon, Portugal; 5 ICT/UNIFESP, Instituto de Ciência e Tecnologia, Universidade Federal de São Paulo, São Paulo, Brazil; 6 IPO Lisboa, Instituto Português de Oncologia Francisco Gentil, Lisbon, Portugal; 7 ITI/LARSyS, Lisbon, Portugal

**Keywords:** Extended skills training in dentistry, Restorative dentistry

## Abstract

To test and evaluate the second installment of DENTIFY, a virtual reality haptic simulator for Operative Dentistry (OD), on preclinical dental students, by focusing on user performance and self-assessment. Twenty voluntary unpaid preclinical dental students, with different background experience, were enrolled for this study. After the completion of an informed consent, a demographic questionnaire, and being introduced to the prototype (on the first testing session), three testing sessions followed (S1, S2, S3). Each session involved the following steps: (I) free experimentation; (II) task execution; S3 also included (III) completion of questionnaires associated with the experiment (total of 8 Self-Assessment Questions (SAQ)); and (IV) guided interview. As expected, drill time decreased steadily for all tasks when increasing prototype use, verified by RM ANOVA. Regarding performance metrics (Comparisons by Student’s t-test and ANOVA) recorded at S3, in overall, a higher performance was verified for participants with the following characteristics: female, non-gamer, no previous VR experience and with over 2 semesters of previous experience of working on phantom models. The correlation between the participants’ performance (drill time), for the four tasks, and user self-assessment evaluation, verified by Spearman’s rho analysis, allowed to conclude that a higher performance was observed in students who responded that DENTIFY improved their self perception of manual force applied. Regarding the questionnaires, Spearman’s rho analysis showed a positive correlation between the improvement DENTIFY inputs on conventional teaching sensed by students, also enhancing their interest in learning OD, their desire to have more simulator hours and the improvement sensed on manual dexterity. All participating students adhered well to the DENTIFY experimentation. DENTIFY allows for student self-assessment and contributes to improving student performance. Simulators with VR and haptic pens for teaching in OD should be designed as a consistent and gradual teaching strategy, allowing multiplicity of simulated scenarios, bimanual manipulation, and the possibility of real-time feedback to allow for the student’s immediate self-assessment. Additionally, they should create performance reports per student to ensure self-perception/criticism of their evolution over longer periods of learning time.

## Introduction

Preclinical teaching in Operative Dentistry (OD) focuses on the development of manual dexterity, which is a keystone clinical skill exerted on a daily basis. Manual dexterity entails the development of motor skills inherent to the acquisition of necessary tactile complexity. An example consists of performing dental cavities with precision in tissues with different physical properties and, challenging enough, present unique resistance to the progression of cutting instruments applied on them.

The most used educational strategy in OD is based on 2 phases^1^: Phase (1) theoretical schematic representation of the procedures to be performed; and Phase (2) training on human models mimicking a real clinical scenario in a simulated environment. It is a goal of the simulation replay to reduce the discrepancy between training in an artificial pre-clinical environment and the real clinical environment. Historically, several methods have been used to promote the simulation and development of delicate manual movements inherent in OD, ranging from the use of real human extracted teeth^2^ to what is considered the cornerstone of pre-clinical teaching in OD: the phantom heads. Introduced in 1894^3^, these are three-dimensional fabricated models in which the student can replicate hypothetical clinical procedures by means of verbal and visual theoretical instructions in a simulated physical environment. Despite their undeniable historical importance, several issues have been pointed out pertaining to their use, namely the lack of realism and the potential subjectivity of the evaluation criteria applied to grade the works performed on them^4^, as well as reduced variability of the simulated environment, in addition to the fact that this assessment can be time-consuming and hurt the student/teacher ratio leading to less time to assist students training^5^.

Virtual reality (VR) with haptic capacities shows up as a promising technology for OD training. VR refers to technology that codes and compiles computer generated, multisensory data to be perceived by users as alternative realities, allowing them to immersively interact with simulated tasks, events or scenarios frequently using head mounted displays. Haptics is defined as the scientific discipline that studies the application of touch (tactile) sensation and control to interaction with computer applications^6^. Head mounted displays and Haptic Technology have been increasingly used in medical training and education bringing teaching simulation closer to clinical reality^7,8^. In Dental Education, several simulators have been developed for Periodontology^1^, Endodontics^9^, Oral Surgery^10^, Prosthodontics^11^ and Anesthesia^12^, yet for OD there is still a gap, and the use of VR and haptic pens has shown promising preclinical teaching results^13–16^ due to its positive impact on accelerating the learning curve and both manual dexterity and motor skills. Moreover, evaluation evidence indicates the usefulness of the haptic simulators in early dental training^15^. They complement the existing phantom head simulators by offering qualitatively different features^17^. There are several studies pertaining to the advantages that simulators add in sensory feedback, which provides better performance in real preparation of virtual enamel and dentin cavities, as well as those that unlimited repetition allowed by VR simulators and haptic pens have on the acquisition and development of psychomotor skills without the need for additional teaching staff^18–21^. A greater number of repetitions allows for faster development of motor skills and manual dexterity competences needed for OD^22^. Both practice through repetition and self-assessment lead to better performance in OD^23^. The models in use for teaching in dentistry are the so-called part-task trainers, ie, a model used to train one particular task and, therefore, to practice and improve one particular skill. In a way, these can be considered modular components of teaching/training which, by acceptance of a partial simulation, when combined, allow for adaptation of the total training system.

The introduction of novel digital technologies for OD teaching is, in 2023, an extensively studied subject. Digital solutions to OD teaching have already been previously tested and compared regarding traditional teaching methods and if, on one hand, the use of VR improves the satisfactory performance of students^15^, on the other hand, although digital methods can be considered as a paradigm shift on dental undergraduate teaching, the human tutor factor cannot be disregarded even when both classical and digital methods show comparable results^24^. The decision on the timing of introducing digital technologies in dental teaching should be, therefore, taken with enormous sensitivity. In this newer version, besides improving the overall aesthetics of the previous version, DENTIFY II includes indirect vision via a virtual mirror, it allows for training virtual dental cavities in both individually isolated teeth or in teeth placed in the virtual mandible, as well as more system metrics for real time evaluations and self assessment..

In context with the aforementioned advantages resulting from exposing dental students to immersive VR haptic simulators (already demonstrated by other commercially available simulators, e.g., Simodont$\circledR$, Virteasy$\circledR$) we developed DENTIFY, a pedagogical multimodal immersive educational simulator, with the aim of recreating the cavity preparation phase inherent to OD in a virtual environment^25^. In this study, we present the second installment of the prototype. Combining a haptic pen, VR headsets, dental turbine drilling sounds, patient-specific teeth models and a laptop, DENTIFY aims to be a pedagogical tool applicable in dental students’ preclinical training environment. The present DENTIFY version was updated and improved, after being previously tested by OD teachers^26^. The aim of this work is to test and evaluate the current version of the DENTIFY prototype on undergraduate (preclinical) dental students, by focusing on user performance and self-assessment.

## Materials and methods

### Concept

DENTIFY is an OD simulator that resorts to VR and haptic pen technology. It places the student/user in an immersive virtual environment, composed of visual, auditory and haptic stimuli. It combines a haptic pen, VR goggles with audio output, turbine and drilling sounds, virtual dental models of isolated teeth and complete dental arches. In order to promote realism of the virtual environment immersion, the haptic pen grip has been replaced by a printed model of a dental turbine. It is intended to be a pedagogical tool for training and teaching the cavity preparation phase in OD, aimed at students in preclinical training for the purposes of improving the student’s manual dexterity and three-dimensional spatial manipulation, increasing the number of repetitions per training session, and, additionally, providing a set of metrics corresponding to each use, per user. The simulator also allows for users to save their data per user and per simulation, thus allowing the user to compile a graphical progressive evaluation. Exposing the student to DENTIFY’s metrics results aims to stimulate self-assessment and self-criticism, as well as the perception of the user’s own learning curve on the grounds of objective and measurable parameters.

As in the previous version^25^, different virtual tissues have different degrees of resistance to in depth progression of the virtual drill. More specifically: virtual enamel offers high resistance to progression; virtual dentin offers intermediate resistance; virtual caries offer low resistance; and no resistance to progression when reaching the virtual pulp. There is the option of defining the virtual cavity boundaries or not. For this study, all tasks had defined virtual cavity boundaries.

**Figure 1:**
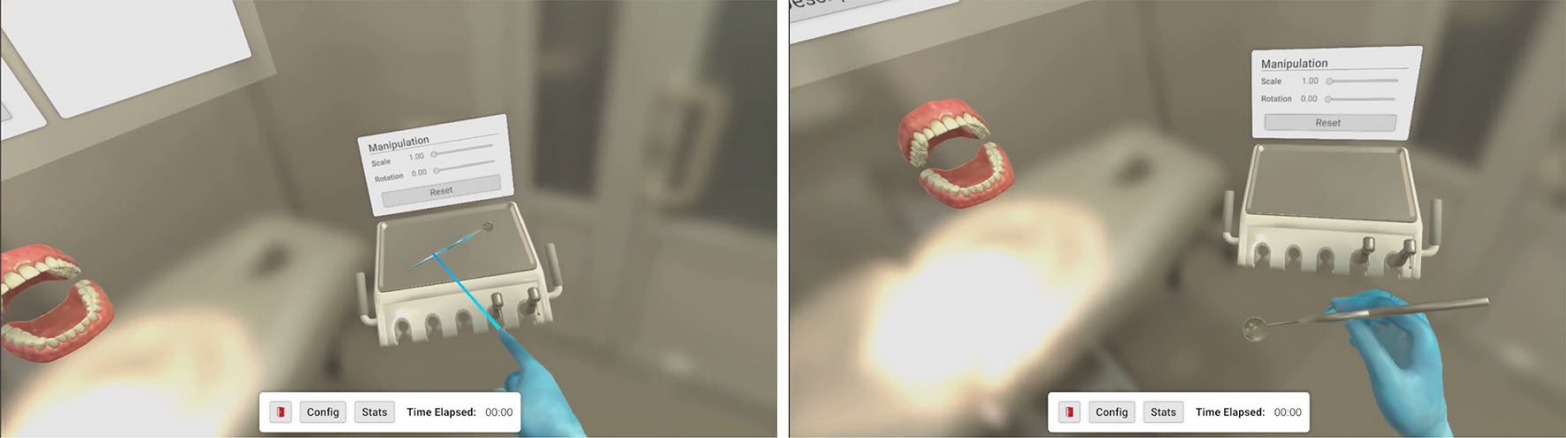
Tool selection process: using the controller, the user points their hand towards the instrument tray and at their desired tool. The selected tool is highlighted in blue and upon pressing the trigger is equiped in the user’s hand. The user may press the cancel button to release the tool.

The version presented in this study presents several add-ons to the previously published version, namely: allowing for more than one type of tooth cavity; realization of virtual cavities in both isolated virtual teeth or in their position in the virtual arch; the possibility of zooming on both isolated virtual teeth and complete virtual arches; indirect vision (including customization of the virtual mirror’s size and, therefore, the depth of field and the dimensions of the reflected image (Fig. 1); and, as mentioned above, allowing for the collection of data pertaining to individual use, thus enabling an objective, numerical and measurable evaluation. Additionally, the new version of DENTIFY allows for three-dimensional manipulation of the individual tooth or virtual arch; allows for the selection of 4 types of virtual spherical drills (in this study, only one virtual drill of equal size was allowed to be used to standardize all users and tasks); and informs the student of the proximity and injury of virtual structures of the used tooth or neighboring teeth, during the simulation.

### Visual, haptic, and auditory components

For the visual component, we resorted to a pair of Oculus Quest 256GB, which was strapped to the user’s head while seated at a desk. Regarding the haptic component, touch feedback was provided by a haptic pen by 3D Systems Touch Haptic Device to perform cavity preparation tasks. Integrated sound speakers of the Oculus Quest provided audio feedback.

We recorded real sound from high rotation instruments in contact and without a dental structure to incorporate into the simulator. Following the recording, we applied a low-pass filter, whenever deformation occurred, to achieve a different sound that’s closer to the real one during the wear phase.

### Apparatus and software

**Figure 2:**
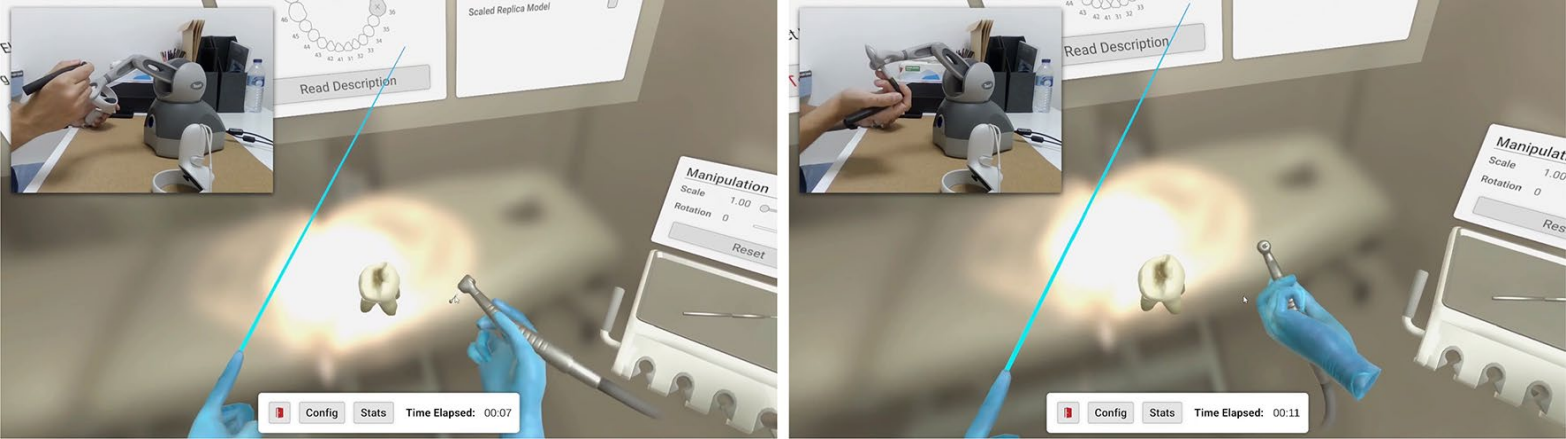
Haptic Pen and VR Controller combined workflow: using the haptic pen with their dominant hand, the user moves the drill, feeling force feedback and vibration. With their other hand, the user holds a regular VR controller that can be used to manipulate the environment, interact with the UI or operate auxiliary tools.

Our setup consisted of a laptop computer (Intel$\circledR$Core TM i7.8750H CPU, 2.20GHz 2.21GHz Processor, 512GB RAM, 1TB HDD, NVIDIA GeForce RTX 2060) running Windows 10 x64 bits. DENTIFY was developed in Unity3D (version 2021.1.0) using the C# programming language. Integration with Virtual Reality was done using the XR Interaction Toolkit plugin. To integrate the haptic pen with the simulator, we resorted to the Openhaptics$\circledR$Unity Plugin, developed and maintained by 3D Systems . This integration, paired with adjustments to the resistance offered and to the vibration level, makes it possible for users to feel touch while manipulating and deforming virtual objects. The replication of the use of a turbine was possible due to enough movement and rotation freedom allowed by the haptic device and a feedback force of up to 3.3 N, provided by the admittance-controlled device PHANToM Omni (SensAble Technologies)^26^. This feedback force was activated whenever the virtual drill collided with the virtual tooth. To detect collision we used a simple ray casting technique (Ray/Triangle Mesh Intersection): the drill emits a ray from its tip (raycast technique); once the ray intersects a triangle of the tooth mesh, and if the distance between the drill and the mesh is below a small threshold, then the mesh is instructed to deform at the point of intersection and the audio emitted by the turbine switches to the sound of dental tissue being drilled. To make the participants feel more immersed in the training experience, a 3D-printed replica of a dental turbine was attached to the haptic pen (Fig. 2).

### 3D image data

**Figure 3:**
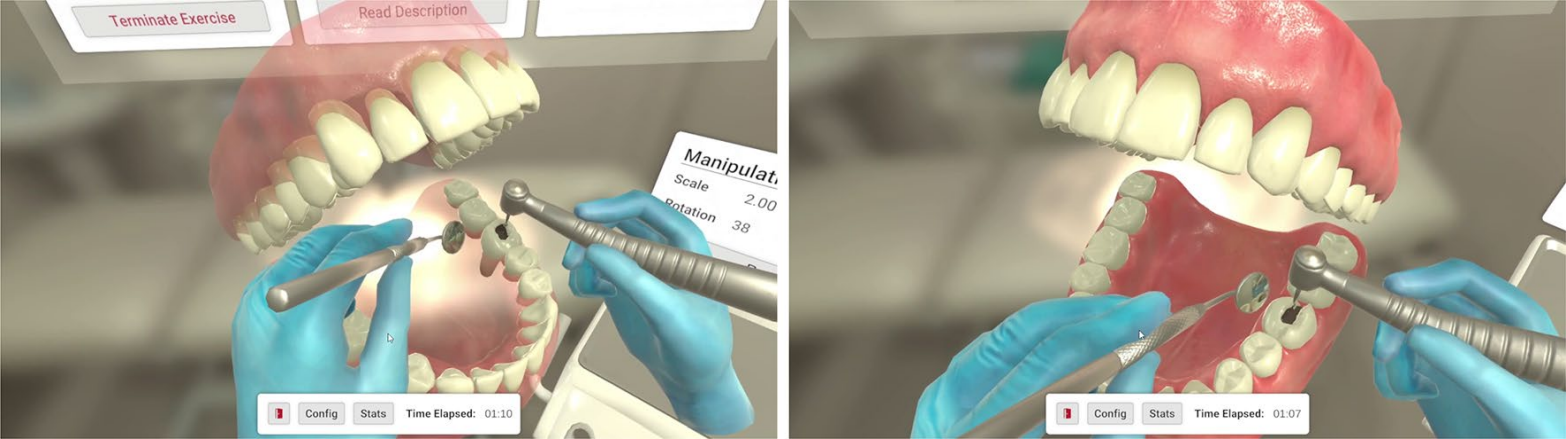
Different cavity types supported: on the left, a class 1 cavity, and on the right, a class 2 cavity. The denture model is scaled to double its original size and the drilling is executed with the help of a mirror for extra visibility. Additionally, the opacity of the Gums was turned down so the user could clearly see the root of the tooth.

The virtual molar that was used on all the exercises was the result of an intra-oral scan (using 3M True Definition, 3M Oral Care) of a healthy tooth freely donated to the Human Tooth Bank at Egas Moniz Dental Clinic. To obtain scans for different types of cavities (Class I and II), that same tooth was prepared using a dental turbine and a spherical burr, starting with a Class I cavity, and then expanding the compromised area to the interproximal surface to create a Class II cavity (Fig. 3). Each of the cavity preparations was scanned using the same method as the original tooth.

These scans were further manipulated using a 3D modeling open-source software (Blender, Amsterdam, Blender Foundation) and saved as STL files. The final models were of great detail and accuracy and consisted of approximately 30,000 vertices.

We were able to model three tooth variations: a healthy one, a tooth with a cavity on one surface and a tooth with a cavity on two dental surfaces. Then, by overlapping the models with cavities with the healthy one, we were able to extract the limits of the cavities, which allowed us to show these limits on the healthy tooth.

The images are screenshots taken from the software developed solely by the authors for the purpose of this study. All the materials used in this software were created by us with the exception of the teeth and gums model which were published under a CC0 license and thus do not legally require attribution.

### Simulation environment

The virtual simulation environment is provided by the VR headset and its controllers in conjunction with the haptic simulation device, all connected to the same laptop. There are some configurations available for the environment, such as the number of teeth in the simulation (either one tooth or a full set of teeth), the type of cavity (Class I, affecting one dental surface or Class II, involving two dental faces), and the user’s dominant hand. These configurations are all done by the instructor.

**Figure 4:**
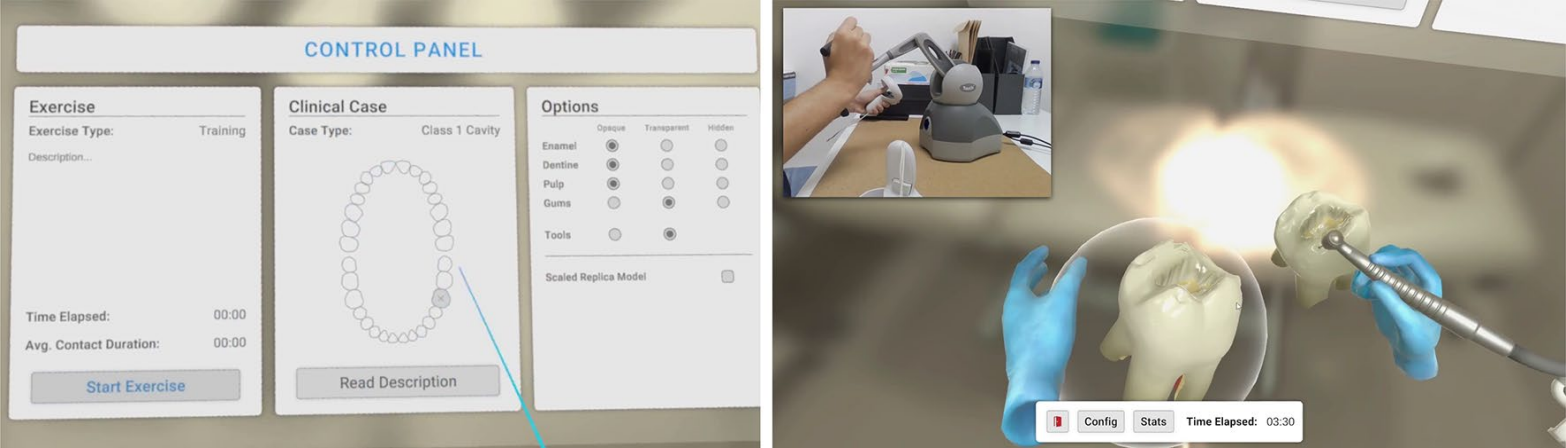
Optional settings: in training mode, the user is given a wide range of settings and information to complement the exercise. A text, visual and spoken description of the clinical case, metrics along with visibility options. The user may start the exercise whenever they are ready to begin. Scaled Replica model: optionally, the user can manipulate a large copy of the tooth in their non-dominant hand for referencing during the exercise.

Once the user moves to the execution stage, a voice starts reading the exercise description shown on a virtual panel in front of the user. This description depends on the type of exercise selected. To begin the exercise, the user must click the OK button, which displays a timer and reveals the tooth to be deformed and the turbine. To grab the turbine, manipulate the tooth or interact with the panel, the user can use the controller in their non-dominant hand. The manipulation of the tooth allows modification of its rotation, position, and scale (Fig. 4). On their non-dominant side, the user finds a tool shelf with four types of drills with different diameters and shapes. These models were created using Blender.

When the user is ready, they should click the Start Exercise button, which starts the timer and allows the drilling to begin. The tooth is deformed by making contact between the tip of the drill and the surface of the tooth, which is achieved by handling the haptic pen as if it were a real dental turbine. Whenever the user feels the deformation they performed matches the one described on the assignment, they should click Terminate Exercise. The timer then stops, and the haptic device is deactivated.

### System metrics and task assessment

DENTIFY allows for the collection of various performance data pertaining to each user, per use. The collection of this data aims to establish an evolutionary curve of the student’s learning over time, by comparing each session’s results at different points in time. Additionally, it allows the student’s performance to be broken down into numerically objective and measurable data, thus providing the student with the possibility of self-assessment on each set criterion. The current version of DENTIFY has several evaluation parameters, and the following were considered: task execution speed (simulation elapsed time) and drill-tooth contact time (drill time). Additionally, it allows distinguishing three different types of contact between drill and virtual model: normal (less than 2 s), prolonged (between 2 and 4 s), and very prolonged (more than 4 s). These contacts can be measured independently in virtual enamel and dentin. During the task execution, the student is informed when contacts are prolonged and very prolonged by audio and chromatic stimuli. Similarly, DENTIFY informs the student of iatrogenic contacts with adjacent teeth (in simulations with the virtual tooth in its position in the arch), inadvertent contacts with the gingiva, and close pulpal contacts. DENTIFY aims to take full use of the full potential of self-assessment using 3 essential strategies: (1) by allowing users to measure the final results after a single task execution; (2) by comparing their evolution through time, thus establishing a growth curve; and (3) by including a digital real-time evacuation system (intra-operatory), namely the 3 level contact time between the virtual drill and the virtual model.

### Participants

Twenty preclinical (3rd year) dental students were enrolled for this study. All participants agreed to participate voluntarly. None of the members of the research team had direct teaching responsibilities on the participants. Participants mean age was 22.0 years old and it was comprehended between 22 and 36 years. The group consisted of 14 (70%) females and 6 (30%) males. Regarding the previous experience with phantom heads and plastic tooth models, fifteen participants (75%) reported having two semesters of experience in performing dental cavities on phantom models as part of their OD classes while five participants (25%) reported having more than two semesters of experience. Additionally, 25% of the participants reported having performed between 10 and 15 dental cavities in flask teeth (i.e., models of plastic teeth inserted into plastic maxillae and/or mandibles), 25% reported having done between 15 and 20 cavities, 25% between 20 and 25 cavities and 5 participants (25%) reported having done more than 25 dental cavities in plastic models. When considering VR and Haptic Technology (HT) experience, 19 participants (95%) reported never having used a haptic pen, and 4 participants (20%) stated having previously experimented with VR. Five participants (25%) identified themselves as gamers.

### Tasks and procedures

After the completion of informed consent and a demographic/user profile questionnaire, followed by a habituation task, participants were asked to perform three testing sessions (S1, S2, S3). Each session involved the following steps: (I) free experimentation; (II) task execution; and S3 also included (III) guided interview. The demographic/user profile questionnaire was conducted during the first session, prior to the free experimentation. Google Forms was used to collect the following data: (i) Gender; (ii) Age; (iii) Do you have experience with plastic models?; (iv) Is this the 1st year that you are using plastic models?; (v) How many cavities have you performed in plastic models during the preclinical Operative Dentistry teaching?; (vi) Have you ever experienced Virtual Reality?; (vii) Have you ever experienced Haptic Technology); and (viii) Are you a gamer?.

The guided interview was not conducted verbally. Instead, each participant was given a Google Docs document to answer two types of questions: free responses, quantitative analysis questions using the Lickert scale and close-ended questions: (1) The free response questions were: (i) What is your opinion on training with phantoms/plastic models?; and (ii) What do you consider to be the limitations of the current teaching method with phantoms and plastic models? (2) The quantitative analysis questions graded using a 5-point Likert scale were: (i) Did you feel that experimenting with the simulator contributed to improving traditional teaching/learning in preclinical Operative Dentistry?; (ii) Do you find that the simulator increased your interest in Operative Dentistry?; (iii) Would you like to have more hours of simulator training?; (iv) Do you consider you have a better manual perception of the force to apply in a dental turbine after training with DENTIFY?; (v) Do you think that training with the DENTIFY simulator can contribute to your 3D visual perception of the dental cavities?; (vi) In your experience, can the evaluations of some phases of the procedures in plastic models be subjective (two tutors evaluating the same result with different grades)?; (vii) Do you think your preclinical Operative Dentistry training can be more independent using DENTIFY?; (viii) Do you consider that a longer use of DENTIFY can further improve your manual dexterity?; (ix) Do you consider that the extended contact warnings contribute to your perception of the iatrogenic risk of overheating when performing a cavity? (3) The close-ended questions were: (i) What type of exercise did you find most relevant to your learning using DENTIFY?; and (ii) In which exercise using DENTIFY did you find the use of the virtual mirror most helpful?

The study was conducted in three different experimentation sessions at different points in time. In each session, the student performed four tasks: (I) Black’s Class I cavity on an isolated virtual tooth (left lower first molar) in the simulator (C1); (II) Black’s Class II occluso-distal cavity on an isolated virtual tooth (left lower first molar) in the simulator (C1I); (III) Black’s Class I cavity on a virtual tooth (left lower first molar) in arch, in the simulator (C2); (IV) Black’s Class II occlusal-distal cavity on a virtual tooth (left lower first molar) in arch, in the simulator (C2I).

The free trial period was no longer than 5 minutes per user and only ran for the first test session.

#### Hygiene and safety

Physical contact is required to use the simulator. In the post COVID-19 pandemic period, its use by multiple students required personal protection measures to ensure hygiene and biosafety conditions, namely the use of a KN-95 mask, disposable or autoclavable cap and thorough disinfection between participants.

#### Ethics approval

The study was approved by the Ethics Committee of the Egas Moniz - Cooperativa de Ensino Superior, CRL, on April 28, 2022.

All methods were performed in accordance with the relevant guidelines and regulations.

### Data analysis

The collected data were statistically analyzed by using descriptive and inferential methodologies, through IBM’s SPSS Statistics v.28 software. A 5% significance level was set in all inferential analyzes.

## Results

The participants’ performance results (drill time), as a function of the experimentation sessions are displayed on Table 1. As expected, drill time decreased steadily for all tasks with increasing prototype use. However, the decrease was not statistically significant for task C1 (p = 0.071).

**Table 1:** Drill time (s) (presented as mean standard deviation) for the considered tasks, as a function of the consecutive experiment sessions (S1–S3).

Task	S1	S2	S3	p*
C1	104.9 (27.0)	91.1 (28.9)	88.2 (33.3)	0.071
C1I	170.1 a (56.4)	102.5 b (34.6)	101.2 b (36.0)	<**0.001**
C2	146.9 a (58.5)	136.1 a (38.9)	112.4 b (40.7)	**0.005**
C2I	170.1 a (41.7)	140.1 b (48.2)	136.6 b (42.4)	<**0.001**

*RM ANOVA. statistically significant differences (p < 0.05) are denoted in bold. Different letters indicate significantly different means.

The performance results, recorded at experiment session S3, as a function of the participants characteristics and previous experience are displayed in Table 2. Overall, a higher performance is identified for participants with the following characteristics: female, non-gamer, no previous VR experience and with over 2 semesters of previous experience of working on phantom models. Significant differences are notably most present on task C1.

**Table 2:** Drill time (s) (presented as mean standard deviation), at experiment session S3, for the considered tasks vs. participants characteristics/previous experience.

		n (%)	C1	C1I	C2	C2I
Gender	Female	14 (70)	81.9 (30.1)	92.9 (31.2)	103.3 (34.6)	**118.9 (36.2)**
	Male	6 (30)	103.1 (38.4)	120.5 (41.9)	133.7 (49.2)	**161.3 (43.6)**
Previous experience in performing dental cavities on phantom models (no. semesters)	2	15 (75)	**97.7 (32.5)**	101.7 (33.5)	117.4 (42.1)	139.4 (41.6)
	>2	5 (25)	**59.7 (14.5)**	99.8 (47.2)	97.4 (36.2)	108.2 39.7 ()
Previous experience with plastic tooth models (no. of dental cavities)	15-Oct	5 (25)	83.0 (35.5)	98.7 (11.7)	116.4 (51.7)	127.2 (35.2)
	15–20	5 (25)	78.5 (32.2)	99.7 (46.8)	99.9 (13.5)	127.5 (34.1)
	20-25	5 (25)	95.4 (45.8)	106.6 (55.3)	102.9 (45.5)	146.4 (74.4)
	>25	5 (25)	96.1 (24.0)	99.9 (27.0)	130.6 (47.0)	125.3 (15.0)
Previous experience with VR	Yes	4 (20)	**115.9 (42.1)**	124.1 (54.1)	123.4 (53.1)	156.7 (61.4)
	No	16 (80)	**81.3 (28.2)**	95.5 (29.7)	109.7 (38.7)	125.3 (36.2)
Gamer	Yes	5 (25)	**110.9 (38.4)**	109.9 (53.2)	95.7 (29.2)	146.8 (57.0)
	No	15 (75)	**80.7 (28.9)**	98.3 (30.3)	118.0 (43.3)	126.5 (37.4)

*Comparisons by Student’s t-test and ANOVA (for previous experience with plastic tooth models). Significantly different means (p < 0.05) are denoted in bold.

The user self-assessment was evaluated by the answers to eight questions (SAQ1–SAQ8), based on a 1–5 Likert-scale. Results are presented in Table 3.

**Table 3:** User self-assessment evaluation.

User self-assessment questions (SAQ)	Median (IQR) *	Min.-Max.	C1	C1I	C2	C2I
SAQ1—did using the simulator contribute to improving conventional OD teaching?	4 (1)	3–5	0.177	0.139	0.353	0.282
SAQ2—do you think using the simulator enhanced your interest in learning OD?	4 (2)	3–5	0.195	0.132	0.139	0.378
SAQ3—would you like to have more simulator hours?	4 (1)	2–5	− 0.008	0.017	0.000	− 0.158
SAQ4—did using DENTIFY allowed you to have a better perception on the manual force to apply?	4 (2)	2–5	0.375	− 0.046	**0.540**	0.385
SAQ5—did using the simulator contribute to improving your 3D perception of dental cavities?	5 (1)	3–5	− 0.383	0.155	− 0.016	− 0.021
SAQ6—do you consider that using DENTIFY allowed you a more independent preclinical training?	4 (1)	2–5	− 0.009	0.002	− 0.001	0.154
SAQ7—do you consider that a more prolonged exposure to DENTIFY could contribute to improving your manual dexterity?	4 (1)	1–5	− 0.035	− 0.188	0.172	0.155
SAQ8—do you consider that the prolonged contact warnings were beneficial for your personal perception of the overheating risks when performing a dental cavity?	5 (0)	3–5	− 0.029	− 0.182	0.237	0.094

* *IQR* interquartile range. Correlation between performance (drill time (s)) and answers to the user self-assessment questions (SAQ). Statistically significant correlations (p < 0.05) are denoted in bold.

The correlation between the participants performance (drill time), for the four tasks, and user self-assessment evaluation are presented in Table 3. It is worth mentioning the statistically significant positive correlation between (SAQ4) and a higher performance for task C2 (rho = 0.540, p = 0.014).

The correlation between the answers to the user self-assessment questions are presented in Table 4. Several significant positive correlations are identified, namely between answers to SAQ1 and SAQ2 (rho = 0.480, p = 0.032), SAQ3 (rho = 0.567, p = 0.009) and SAQ7 (rho = 0.508, p = 0.022). Additionally, the answers to SAQ2 are also significantly positively correlated with SAQ3 (rho = 0.478, p = 0.033) and SAQ6 (rho = 0.488, p = 0.029).

**Table 4:** Correlation between the answers to the user self-assessment questions (SAQ).

	SAQ1	SAQ2	SAQ3	SAQ4	SAQ5	SAQ6	SAQ7	SAQ8
SAQ1	–	**0.480**	**0.567**	0.232	0.269	0.226	**0.508**	0.316
SAQ2	–	–	**0.478**	0.012	0.332	**0.488**	0.390	0.259
SAQ3	–	–	–	− 0.398	0.281	0.353	0.322	0.179
SAQ4	-	–	–	–	− 0.017	− 0.235	0.051	0.343
SAQ5	–	–	–	–	–	0.185	0.128	0.378
SAQ6	–	–	–	–	–	–	0.629	0.419
SAQ7	–	–	–	–	–	–	–	0.352
SAQ8	–	–	–	–	–	–	–	–

*Statistically significant correlations (p < 0.05) are denoted in bold.

## Discussion

The transition from pre-clinical teaching to clinical teaching in Dentistry is a unique moment in the student’s life due to the vast array of technical and personal difficulties that the student must overcome. It is a challenging phase that can be considered one of the most critical moments in the construction of a professional identity, both technically and personally. The act of dentistry, in OD specifically, requires a millimetric tactile sensitivity and manual dexterity that allows for the removal of diseased tissue without damaging healthy tissue, under risk of iatrogenically damaging delicate healthy dental tissues. One of the main prerogatives of pre-clinical training is to reduce the transitional shock between simulated and real models. Simulated models should therefore be as close as possible to the clinical setting in order to minimize this transition. The authors consider that models based on and/or obtained from real tissues tend to reduce this discrepancy. The closeness between simulation and reality is one of the possibilities conferred by VR and haptic technology, both for the preparation of the medical act itself, and just as importantly, for teaching. Allowing objective assessments; increasing the repetition and number of training hours; increasing the degree of realism of pre-clinical scenarios; making the preclinical student aware of the risks of medical error and iatrogenesis; these are all dimensions added to teaching with the use of these technologies, possibly placing the student further along the learning curve. However, putting these simulator-embedded strategies to use in the teaching of operative dentistry, by itself, is not enough. Although these scenarios may be graphically irreproachable, they should be equipped with concrete measures of immediate evaluation and compared over time. Breaking down evaluation into objective parameters allows for the dissection of the student’s self-criticism on the different points that constitute a clinical protocol, and therefore their awareness of its limitations and iatrogenic risk factors.

Studies^27^ have demonstrated that head-mounted displays can have an effect on user performance during task execution and visual discomfort. Head-Mounted Displays’ discomfort has been evaluated through Simulator Sickness Questionnaires (SSQT), oculomotor scores of Simulator Sickness Questionnaires (SSQO), and Visual Strain Questionnaires scores (VSQ)^27^. Furthermore, visually induced motion sickness (VIMS) or simulation sickness, are not to be underestimated and can be considered as an obstacle to the massification of these technologies^28^. Head-Mounted Displays manufacturers must not underestimate these factors and be aware of them during design product and commercialization. Despite the possibility of current HMD devices inducing simulator sickness, none of the participants of our study reported any situation of cyber sickness (e.g., headaches, visual discomfort, etc).

The exact timing for introducing simulators with VR and haptic pens into the preclinical student’s learning path in OD has been discussed and promising results have been reported when they are exposed to the simulators prior to simulation on plastic models^29^, as it amounts to a complementary strategy to traditional teaching methods^30^.

**Figure 5:**
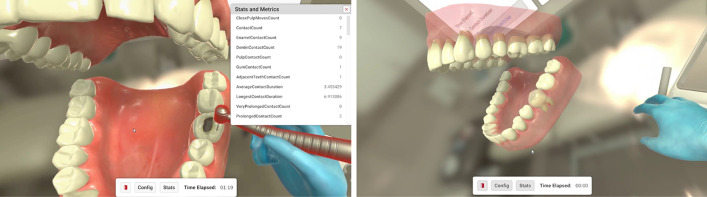
Over the duration of the exercise an array of metrics is collected and saved for analysis. These metrics can be monitored in real time by an examiner on the computer screen. The gums along with every layer of the tooth tissue can be set to translucent or invisible allowing users to easily view inner structures like the dentine and pulp that would otherwise always be obscured.

Unlike other dental virtual reality simulators used in OD, DENTIFY includes the simulation of caries removal on more than one tooth (depending on the degree of difficulty established for the given exercise), isolated or in its position in the dental arch, bimanual handling, and indirect zoomable vision using a virtual dental mirror. It already includes a multisensory stimulation by incorporating visual, tactile, and auditory stimuli in the VR environment. Furthermore, it allows the learner real-time feedback and evaluates task performance on a set of predefined metrics both per user and per use (Fig. 5). This version consists of the consistent natural evolution of the previously presented simulator^25^.

One of the parameters used to evaluate student performance was the progression of task completion time from S1 to S3. There was a general reduction in the time taken to perform all cavities, by all students, both in single tooth and in arches, and in both types of dental cavities (Black’s classes I and II), from the first to the third session, although this was not statistically significant regarding C1. The overall improvement may be attributed to the fact that the students got used to the simulator and, therefore, had greater dexterity in performing dental cavities in a virtual environment as a result of frequent use. The repetition of the task allowed all students to show similar improvements in task completion times. Interestingly, the reduction in execution time between the first and third sessions was more noticeably observed in female students in all tasks performed, especially for C2I. It would be expected that students with more experience in performing cavities on plastic models would show shorter task completion times in the simulator. This relationship was observed. Students with less training time with plastic models showed longer execution times in all tasks, compared to the students with more than 2 semesters of experience in performing cavities in phantom heads, in the preclinical teaching context.

It is relevant and interesting to report that the students that classified themselves as gamers showed no significant reduction in execution times compared to the non-gamers. Interestingly, non-gamer students, in the third testing session, showed significantly better results in the Class I cavity situations in virtual isolated tooth (C1). This result allows us to assume that previous experience with computerized challenges and goal achievement scenarios, as well as frequency and time of experience with computer games, proved to be non-fundamental to the acquisition of manual dexterity in the simulator; in other words, users with no previous gaming experience could expect a performance progression similar to that of gamers. Importantly, and similarly, students without virtual reality experience showed superior drilling times in all tasks performed, in the third testing session. However, no significant differences were detected in the improvement of task execution times between students with prior VR experience relative to students with no prior VR experience for C1I, C2 and C2I tasks. This difference was only found to be statistically significant for task C1.

When comparing gamers vs. non-gamers, and experience with VR vs. no VR experience, the authors consider it possible to attribute these results to the interface design and the specific nature of the task. On the one hand, regarding the virtual environment, the scenarios presented are of such easy accessibility and of intuitive interaction that it allowed non-gamers/non-VR experienced students to encounter little difficulty when navigating and interacting in the virtual scenario, and even present faster task execution. On the other hand, if inexperience in gaming/VR is not a conditioning factor in the results obtained in the simulator, we can infer that it is expected that a gamer/user of VR with no previous education in OD will not show better results than a pre-clinical dentistry student with no gamer and inexperience in VR. The theoretical training process in OD appears to be a limiting factor in simulator performance (not gaming/previous VR experience, per se).

Regarding the use of phantom heads as a pre-clinical teaching method in OD, the users were very clear in pointing out their pedagogical usefulness. However, they also pointed out negative parameters of their use, such as matters of cost; difference in texture between phantom and natural teeth; absence of information about pressure and prolonged (potentially iatrogenic) contacts between drill and model; absence of visual limits of the prepared cavities; absence of notion of overheating of drills; absence of indirect vision. Additionally, 100% of the students mentioned that procedure evaluation in plastic models could be a result of subjective appreciation. On a grading 1–5 Likert-scale, the students considered, with statistical significance, that exposure to the simulator: contributes to improving traditional teaching; has increased their interest in OD; improves manual perception of the force to be applied on the turbine; and decisively promotes perception of dental cavities’ spatial dimensions. The students also mentioned that they would like to have more hours of exposure to the simulator after the 3rd test session. Users additionally considered that simulator training contributes to more independent learning, and that prolonged exposure to the DENTIFY virtual environment may further increase their manual dexterity despite the subjective nature of tactile sensitivity.

It is also relevant to report that 100% of the students pointed out the warnings for prolonged and very prolonged contact as strongly contributing to improving their self-perception of iatrogenic risk during the virtual cavity performance.

Finally, regarding the different exercises performed, 100% of the students considered that the performance of Black’s Class II cavities with the virtual tooth in its position in the arch was the most relevant learning exercise in the simulator, and the exercise during which indirect view of the virtual mirror proved to be most useful. One can infer that the closer simulated scenarios approach to real scenarios, the more usefulness can be attributed to the training strategy.

Regarding guided interviews, 100% of the students responded that evaluation using conventional OD teaching methods may be subjective, i.e., that there can be discrepancies when different teachers evaluate the same task performed by the same student. In this context, they reported to have felt more objectivity using DENTIFY due to the embedded metrics on the simulator. The results of this study support the concepts guiding repetitive and deliberate practice, and the value of self-assessment in achieving better performance on the same task.

Although this study concluded the usefulness of DENTIFY as a VR and haptic pen simulator regarding student performance and assessment, it was conducted in a few weeks. To further assess the pedagogical advantages that simulator immersion can produce, one should consider comparing evaluation scores of both simulator-exposed and non exposed students. Long term studies should reinforce the utility of VR and haptic technology simulation in OD learning.

Despite cost limitations inherent to the use of these technologies, the authors consider that there is a real and present opportunity to universalize their application to teaching, promoting a healthy technological dispute that will, inevitably, not only produce increasingly realistic simulators, but also reduce the cost of these simulators, thus enabling their eventual acquisition for home use, which, in turn, endows teaching with both greater independence and greater frequency of repetition. VR and haptic technology should be introduced globally and gradually and in a standardized manner in the teaching of operative dentistry.

## Conclusions

This study concluded that the DENTIFY simulator provides pre-clinical students with pedagogical tools that may reduce the difficulty of transitioning to the clinical reality of operative dentistry, while honoring the goal of improving student performance, reducing morbidity and increasing predictability.

The simulators with VR and haptic pens for teaching in OD should be designed as a consistent and gradual teaching strategy, allowing multiplicity of simulated scenarios, bimanual manipulation, and the possibility of real-time feedback to allow for the student’s immediate self-assessment. Additionally, they should create performance reports per student to ensure self-perception of their evolution over longer periods of learning time.

## Future work

The use of VR and haptic devices in preclinical learning, not only in OD but also in other fields of Dental Medicine has demonstrated promising results since it has shown to accelerate the acquisition of the motor skills needed in Dentistry. Virtual artificial models of teeth and cavity types have been applied with promising results in a global strategy to decrease the difficulties in the preclinical to clinical transition. The authors consider this to be a most valuable input in promoting the acquisition of manual dexterity, which is fundamental in this field. However, a few difficulties are still present in today’s contemporary simulators. It is the authors’ belief that increasing the degree of realism in the virtual models can, ultimately, further reduce the distance between simulated virtual models and clinical reality. For instance, as we presented in DENTIFY, the use of real teeth for constructing our virtual environment. We intend to place futures focus on including real clinical cases by use of scanning into our simulator. Furthermore, additional hours of work must be put on improving the deformation provoked by virtual drills on virtual models, in order to approach a raw clinical environment. In this context, the preclinical VR dental student must be confronted with a greater number of clinical situations and so more scenarios must be added to simulators, without disregarding the degree of difficulty of these such tasks. a crescent degree of difficulty , which allows for sustained learning, should be considered. Self assessment and self criticism can become powerful learning tools when they are the result of objective performance evaluation through universally accepted metrics. Further consensus is necessary in order to transfer accurate evaluation criteria into dental learning simulators.

VR simulators can be a promising strategy in learning OD. However, their current cost demands a huge financial investment. This technology can only become a real massive presence in dental schools worldwide when production and commercialization prices decrease.

The presented results were very encouraging for the subsequent development of DENTIFY. In addition, the authors hope that these results may serve to stimulate research and other researchers for the purposes of introducing progressively better and more realistic models in dental education. Although momentaneously limited to OD, our main objective is to convert it into a multidisciplinary simulator in Dental teaching, both in undergraduate and postgraduate contextes. Once we’ve finished the Operative Dentistry simulator, we intend to bring other departments closer and involve them in the construction of a full DENTIFY concept. Our domestic implementation plan includes gradually immersing our dental students in the DENTIFY ecosystem by adding different teaching scenarios. DENTIFY is, ultimately, a pedagogically-driven concept with the objective of bringing these most promising technologies in service of our student’s difficulties. We wish for our students to be able to include number of simulation hours and virtual tasks performed (similarly to what actually happens in Aviation), hoping that these enhance their dental skills and, simultaneously, give them a competitive edge when entering the work market.

Our participants were in good physical health and had no relevant issues that could affect the use of VR headsets. Pre-existing physical impairments can be considered to have influence on user performance and, subsequently, experimental results. These must be taken into consideration for these technologies to become generally accessible in dental schools. Visual impairments are normally corrected by spectacles or contact lenses (it may be interesting to explore whether these corrective measures impact performance) and the probability of undiagnosed visual diseases is seldom. This variable should be addressed in future tests (Supplementary Information S1).

## Supplementary Information

Supplementary Information.

## Data Availability

The data that support the findings of this study are available from the corresponding author upon reasonable request.
